# A molecular survey of acute febrile illnesses reveals *Plasmodium vivax* infections in Kedougou, southeastern Senegal

**DOI:** 10.1186/s12936-015-0808-y

**Published:** 2015-07-19

**Authors:** Makhtar Niang, Laty Gaye Thiam, Abdourahmane Sow, Cheikh Loucoubar, Ndeye Sakha Bob, Fode Diop, Babacar Diouf, Oumy Niass, Annick Mansourou, Marie Louise Varela, Ronald Perraut, Amadou A Sall, Aissatou Toure-Balde

**Affiliations:** Immunology Unit, Pasteur Institute of Dakar, Dakar, Senegal; Department of Animal Biology, Cheikh Anta Diop University of de Dakar, Dakar, Senegal; Arbovirus and Viral Haemorrhagic Fevers Unit, Pasteur Institute of Dakar, Dakar, Senegal

## Abstract

**Background:**

Control efforts towards malaria due to *Plasmodium falciparum* significantly decreased the incidence of the disease in many endemic countries including Senegal. Surprisingly, in Kedougou (southeastern Senegal) *P. falciparum* malaria remains highly prevalent and the relative contribution of other *Plasmodium* species to the global malaria burden is very poorly documented, partly due to the low sensitivity of routine diagnostic tools. Molecular methods offer better estimate of circulating *Plasmodium* species in a given area. A molecular survey was carried out to document circulating malaria parasites in Kedougou region.

**Methods:**

A total of 263 long-term stored sera obtained from patients presenting with acute febrile illness in Kedougou between July 2009 and July 2013 were used for malaria parasite determination. Sera were withdrawn from a collection established as part of a surveillance programme of arboviruses infections in the region. *Plasmodium* species were characterized by a nested PCR-based approach targeting the 18S small sub-unit ribosomal RNA genes of *Plasmodium* spp.

**Results:**

Of the 263 sera screened in this study, *Plasmodium* genomic DNA was amplifiable by nested PCR from 62.35% (164/263) of samples. *P. falciparum* accounted for the majority of infections either as single in 85.97% (141/164) of *Plasmodium*-positive samples or mixed with *Plasmodium ovale* (11.58%, 19/164) or *Plasmodium vivax* (1.21%, 2/164). All 19 (11.58%) *P. ovale*-infected patients were mixed with *P. falciparum*, while no *Plasmodium malariae* was detected in this survey. Four patients (2.43%) were found to be infected by *P. vivax*, two of whom were mixed with *P. falciparum*. *P. vivax* infections originated from Bandafassi and Ninefesha villages and concerned patients aged 4, 9, 10, and 15 years old, respectively. DNA sequences alignment and phylogenetic analysis demonstrated that sequences from Kedougou corresponded to *P. vivax*, therefore confirming the presence of *P. vivax* infections in Senegal.

**Conclusion:**

The results confirm the high prevalence of *P. falciparum* in Kedougou and provide the first molecular evidence of *P. vivax* infections in Senegal. These findings pave the ways for further investigations of *P. vivax* infections in Senegal and its contribution to the global burden of malaria disease before targeted strategies can be deployed.

## Background

Malaria is a major infectious disease and remains the main global cause of death in many endemic areas, including Senegal. Success of malaria control interventions has led to a significant decrease of the disease burden. Between 2000 and 2013 malaria mortality rates decreased by 47% globally, and by 54% in sub-Saharan Africa, the region most affected by the disease [1]. Among the five different species of the genus *Plasmodium* (*Plasmodium falciparum*, *Plasmodium vivax*, *Plasmodium ovale*, *Plasmodium malariae*, and *Plasmodium knowlesi*) known to infect humans [2], *P. falciparum* remains the deadliest in Africa [1] and has been the major focus of malaria interventions.

With decreases in the *P. falciparum* burden resulting from successful malaria control interventions [1], attention must be focused on more than 400 million malaria cases due to other parasite species [3]. *P. vivax*, the most widespread of the *Plasmodium* species, is fast becoming a recognized cause of different grades of malaria pathologies along with an increasing trend in complicated malaria cases, thus threatening the prospect of malaria elimination in parts of Africa [4–8].

The presence of *P. vivax* malaria in sub-Saharan Africa has been largely neglected based on the demonstrated correlation between the lack of expression of the Duffy blood group in sub-Saharan African population and the absence of *P. vivax* infections [9, 10]. However recent reports of *P. vivax* infections in Duffy-negative individuals [7, 8, 11], have called for reconsideration of this widely accepted dogma as they strongly indicate that the Duffy status is no longer a barrier to *P. vivax* infection. Moreover, *P. vivax* appears to be more frequent in countries where either it was not present or it was not detected by the available techniques in the past, as is the case of some countries of West and Central [4, 6–8, 11, 12], becoming therefore a major source of concern.

To date, no *P. vivax* infection has been reported in Senegal despite evidence of its presence in many West African countries [4, 13–15]. A molecular survey of circulating malaria parasites species was carried out on samples collected from acute febrile patients as part of a surveillance programme of arboviruses infections in Kedougou region, southeastern Senegal.

## Methods

### Study site

**Figure 1:**
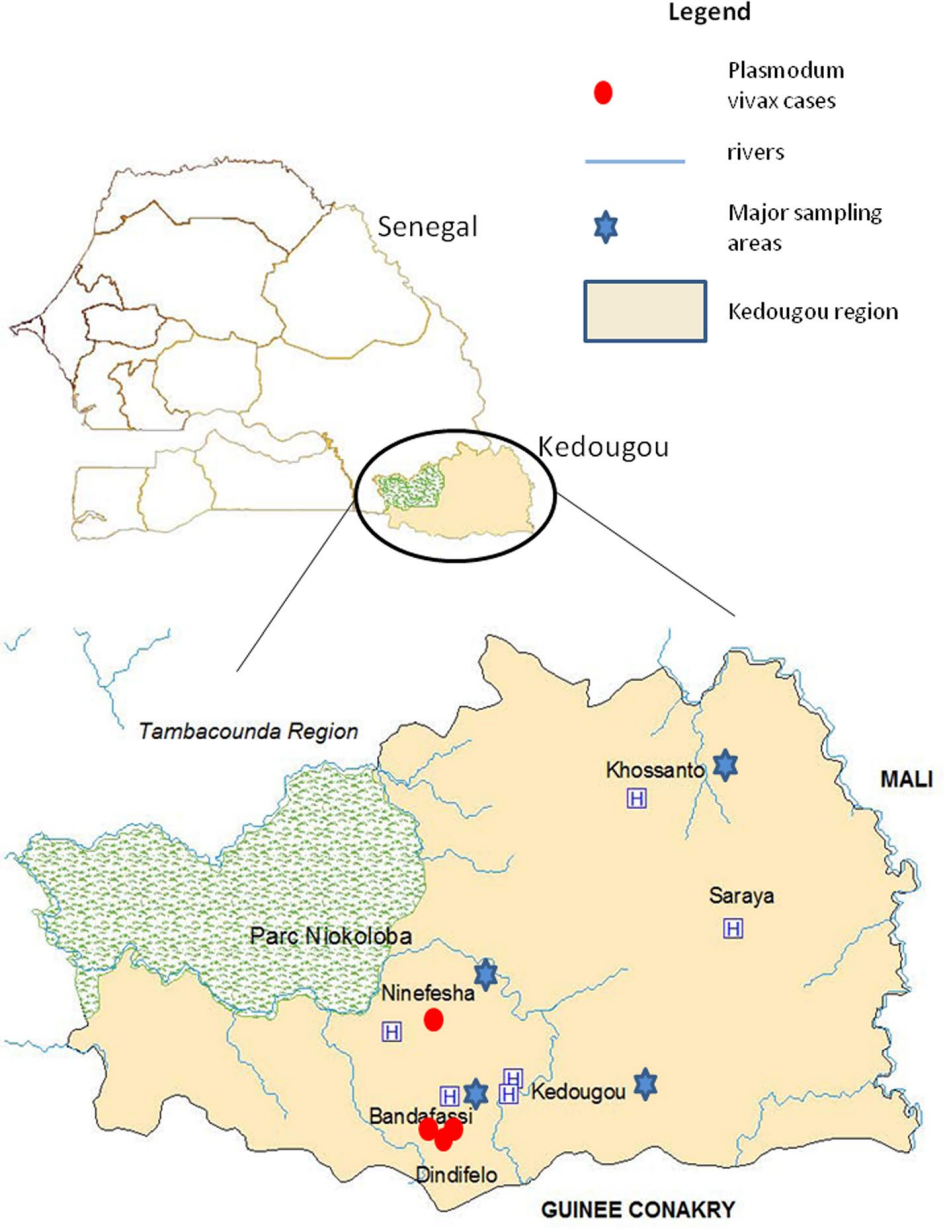
Map of Kedougou region showing villages where the majority of samples were obtained (*blue star*) and origin of the four *Plasmodium vivax* infections (*red circle*).

The study was conducted in the Kedougou region (Figure 1) located in southeast Senegal at the bordering area with Guinea, Mali and Gambia between isohyets 1,200 and 1,300 mm. The climate is Sudano-Guinean with a single rainy season from May to November. The landscape consists of wooded grassland or woodland and dense gallery forest. The fauna is diverse with herbivorous, insectivorous, rodents, and monkeys.

Malaria remains highly prevalent in Kedougou region and transmission is highly seasonal occurring during the rainy season (May–November). In 2014, the Senegalese National Malaria Control Programme reported 25.55% confirmed clinical malaria cases of which 2.73% turned into severe disease [16].

### Population and study design

As part of a genetic diversity study of *P. falciparum* isolates in concurrent malaria-arbovirus infections from patients presenting with acute febrile illness (AFI) in Kedougou, a molecular diagnostic was conducted to discriminate *P. falciparum* from other *Plasmodium* species. This revealed a unique case of *P. vivax* infection in a patient from Ninefesha village. Therefore, additional samples from Ninefesha and nearby villages were retrieved from the sera collection and screened to survey *P. vivax* infections in Kedougou. A total of 263 sera from patients presenting with AFI between July 2009 and July 2013 in the Kedougou region of Senegal were included in this study. Sera were withdrawn from a collection established as part of a monitoring programme of arboviruses in Kedougou region. AFI was defined as ‘any patient older than 1 year with a fever (temperature >38°C) lasting for less than 2 weeks, exhibiting two or more of the following symptoms: headache, myalgia, eye pain, arthralgia, cough, nausea/vomiting, diarrhoea, jaundice, bleeding and neurological signs’.

### Ethical clearance

The study objectives, benefits and risks were explained in French language or local dialects to all participants before inclusion. Written informed consent was obtained from all adults participants and from the parents, or legal guardians of children. The study was examined and approved by the Senegalese National Health Research Committee.

### Molecular detection of *Plasmodium* species

The detection of *Plasmodium* spp. genomic DNA (gDNA) in frozen serum samples has been reported earlier [17, 18]. Genomic DNA isolation of *Plasmodium* parasites was performed using QIamp DNA Mini Kit (Qiagen, Hilden, Germany) according to manufacturer’s instructions. DNA extracted from blood samples of known microscopically confirmed *P. falciparum*, *P. malariae* and *P. ovale*-infected patients were used as positive controls. *P. vivax* gDNA was kindly donated by Dr Ambroise Ahouidi (Le Dantec Hospital, Dakar).

Qualitative detection of *Plasmodium* parasite DNA was based on nested PCR with primers targeting the *Plasmodium* spp. 18S small sub-unit ribosomal RNA (18S ssrRNA) gene as described previously [19]. The primary PCR amplification was performed with *Plasmodium* genus-specific rPLU5 and rPLU6 primers pairs [20, 21] and 1.5 µl of template DNA in a total volume of 25 µl using the GoTaq Green Master Mix protocol (Catalogue no M7113, Promega) according to manufacturer’s recommendations. The nested reaction was performed for the specific detection of *Plasmodium* species using previously described primers pairs rFAL1 and rFAL2 for *P. falciparum*, rVIV1 and rVIV2 for *P. vivax*, rOVA1 and rOVA2 for *P. ovale* and rMAL1 and rMAL2 for *P. malariae* [20, 21]. The genus-specific primary PCR products (1 µl) were used as the template in the species-nested PCR amplification under the same conditions. Nested PCR results were scored as categorical variable (presence vs. absence of amplification). PCR cycling reaction and amplification conditions were as described by Snounou and Singh [19].

### Purification of PCR products, DNA sequencing and analysis

In order to determine the sequences’ identity of the *P. vivax* amplified DNA, the specific bands were extracted from the gel and purified using the QIAquick Gel Extraction Kit as described by the manufacturer (Qiagen^®^). The purified PCR products were then sent to COGENICS for sequencing. For each DNA fragment, sequencing was performed from both the 3′ and 5′ directions (2× coverage). Consensus sequences were generated from the forward and reverse sequences of each sample and used in a BLASTn algorithm against the Genbank database for similarity profile determination.

Sequence alignment and phylogenic trees were performed using Mega 6.06 software. *Plasmodium* spp. small sub-unit rRNA (SSU RNA) sequences were obtained from Plasmodb version 13.0 and Genbank. *Plasmodium vivax*: *P. vivax* Sal1_U030779.1, *P. vivax*_HF945443.1, *P. vivax*_HF945441.1; *P. falciparum*: JQ627152.1; *P. ovale*: JF894411.1; *P. malariae*: GU815531.1. Sequences were analysed by the Neighbour Joining method using 100 bootstrap replicates.

## Results

### Characteristics of the study population

A total of 263 sera from patients presenting with AFI enrolled during arboviruses surveillance in Kedougou were screened in this study for the presence of malaria parasite species. Patients were aged one to 65 years old, the majority originated from the village of Bandafassi (Table 1). The mean age of the population varied between 14 years (range 1–65 years) in Bandafassi and 23 years (range 4–60 years) in Kedougou (Table 1). The sex ratio was in favour of females in Bandafassi and in favour of males in others villages (Table 1).

**Table 1:** Characteristics of the study population

	Kedougou	Bandafassi	Ninefesha	Other	Total
Number	57	149	34	23	263
Percentage (%)	21.67	56.65	12.93	8.75	100
Age					
Mean	23	14	16	21	
Range	[4–60]	[1–65]	[1–62]	[1–50]	
Sex					
Male	31	73	19	13	136
Female	26	76	15	10	127
M/F	1.19	0.96	1.26	1.3	

### Detection of malaria parasite species in patients by nested PCR

**Figure 2:**
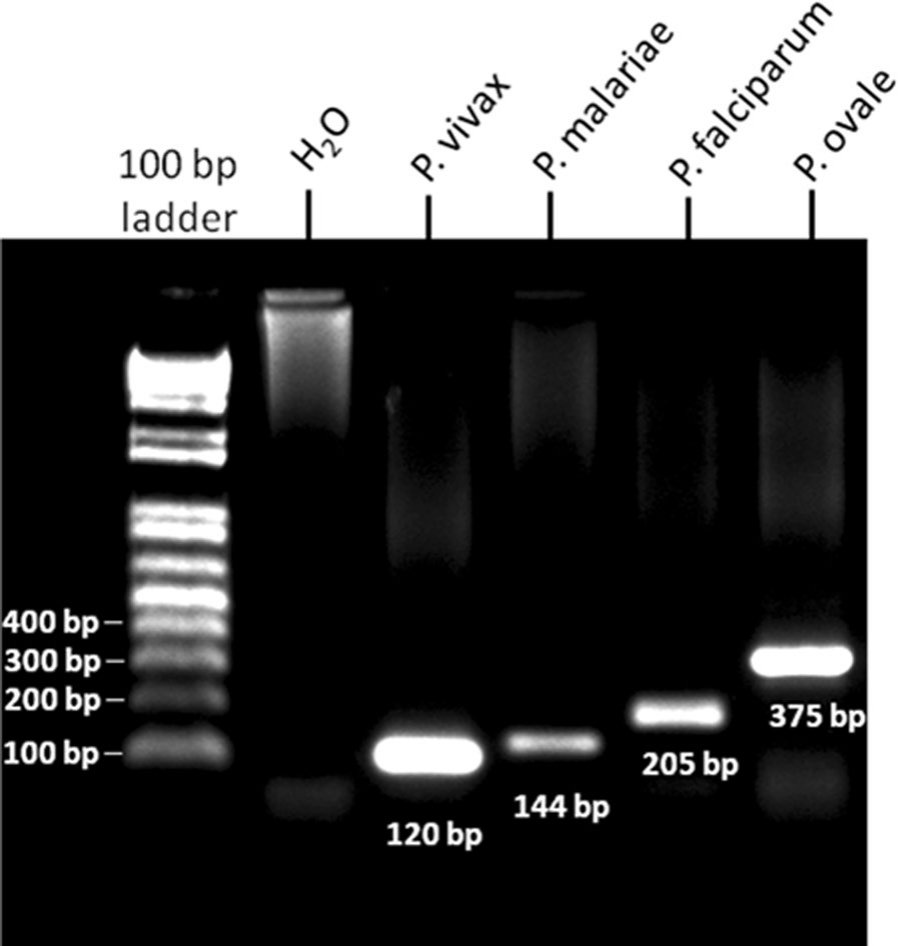
Gel picture showing positive amplification of *Plasmodium vivax* (120 bp), *Plasmodium malariae* (144 bp), *Plasmodium falciparum* (205 bp) and *Plasmodium ovale* (375 bp) 18S RNA gene. 100 bp DNA Ladder was used to determine molecular size.

The four previously diagnosed clinical samples (*P. falciparum*, *P. vivax*, *P. malariae*, and *P. ovale*) and sterile distilled water were used as the positive and negative controls, respectively to validate the nested PCR approach. The presence of amplified DNA products in a patient’s specimen corresponding to *P. falciparum* (205 bp), *P. vivax* (120 bp), *P. malariae* (144 bp) and *P. ovale* (375 bp) was confirmed (Figure 2).

*Plasmodium* genomic DNA was amplifiable by nested PCR from 62.35% (164/263) of the 263 sera screened in this study (Table 2). *P. falciparum* accounted for the majority of infections and was present either as single infections in 85.97% (141/164) of positive samples or mixed with *P. ovale* (11.58%, 19/164) or *P. vivax* (1.21%, 2/164). Nineteen patients (11.58%) were infected with *P. ovale*, all of which were mixed with *P. falciparum* (Table 2). *P. malariae* was not detected in the current survey despite report of its presence in Kedougou (Ndiaye et al. unpublished data). Four patients (2.43%) were found to be infected by *P. vivax*, two of which were mixed infections with *P. falciparum* (Table 2). Three of the four cases of *P. vivax* infections originated from the village of Bandafassi and concerned two females aged 4 and 10 years old and one male aged 15 years. The fourth *P. vivax* case originated from a 9 years old female from Ninefesha village (Table 2).

**Table 2:** Details of single and mixed *Plasmodium* species infections in Kedougou

Site (sample size)	*Plasmodium species*					
	*P. falciparum*	*P. vivax*	*P. malariae*	*P. ovale*	*P. falciparum* *+* *P. vivax*	*P. falciparum* *+* *P. ovale*
Kedougou (57)	42	0	0	0	0	5
Bandafassi (149)	77	1	0	0	2	9
Ninefesha (34)	4	1	0	0	0	3
Others (23)	18	0	0	0	0	2
Total	141	2	0	0	2	19

### Sequences analysis confirmation of *Plasmodium vivax* infections

**Figure 3:**
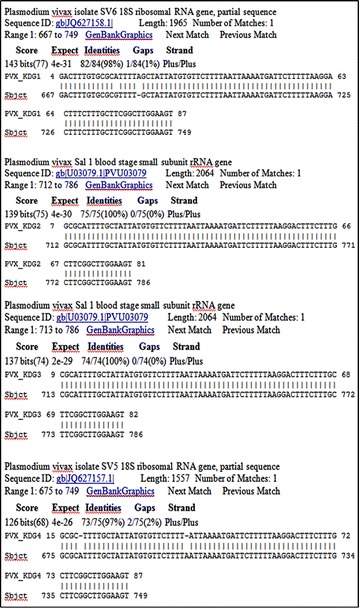
BLASTn output of the sequences generated from the four *Plasmodium vivax*-infected patients. Each of the sequences was queried against the *Plasmodium* GenBank data and number of hits with percentages identities displayed.

To substantiate the PCR assays results on the four incidences of *P. vivax*, DNA sequencing was performed for the four samples for gene specific to *P. vivax*. BLASTn analyses of the DNA sequences generated from the positive *P. vivax* samples confirmed that the *P. vivax* sequences from Kedougou (PVX_KDG1, PVX_KDG2, PVX_KDG3 and PVX_KDG4) matched the Genbank *P. vivax* isolate SV6 18S ribosomal RNA gene partial sequence (ID: JQ627158.1), *P. vivax* Sal1 blood stage small sub-unit rRNA gene (ID: PVU03079) and *P. vivax* isolate SV5 18S ribosomal RNA gene partial sequence (ID: JQ627157.1) by 98, 100, 100, and 97%, respectively (Figure 3).

**Figure 4:**
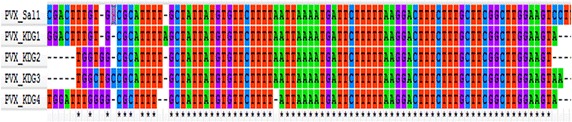
DNA sequence alignment of the four Senegalese *Plasmodium vivax* isolates with the reference DNA sequence of *P. vivax* SAL-1 strain.

In addition, multiple sequences alignment of the four DNA sequences from *P. vivax*-infected samples and reference 18S rRNA gene sequence of *P. vivax* Sal1 strain indicated perfect homology with limited polymorphism between the Kedougou *P. vivax* DNA sequences and the reference *P. vivax* sequence, therefore demonstrating that sequences from Kedougou corresponded to *P. vivax* (Figure 4).

**Figure 5:**
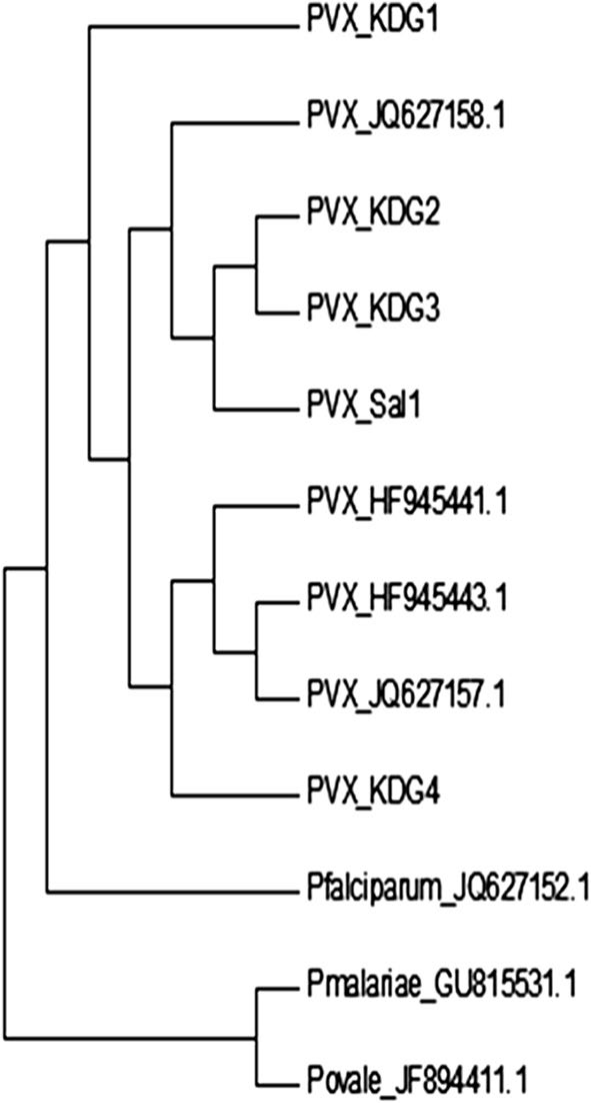
Bootstrapped phylogenetic rooted tree constructed by the Neighbour Joining method for the SSU rRNA sequences of *Plasmodium vivax* isolates from Kedougou. Horizontal branch lengths between nodes correspond to the number of shared derived changes. *Plasmodium* spp. small sub-unit rRNA (SSU RNA) sequences were retrieved from Plasmodb.

Consistent with the existence of different small sub-unit RNA genes in *P. vivax* [22], phylogenetic analysis showed that sequences from Kedougou cluster with SSU RNA of *P. vivax* genes of different isolates and strains while branching-out from sequences of other *Plasmodium* spp. (Figure 4). PVX_KDG1 and PVX_KDG4 were closely related to PVX_JQ627158.1 and PVX_JQ627157.1, respectively, while PVX_KDG2 and PVX_KDG3 clustered with PVX-Sal1 reference strain (Figure 5). These observations are in accordance with the sequences analysis data (Figure 3). Taken together, these results confirmed the presence of *P. vivax* infections in Senegal.

## Discussion

The detection of *P. falciparum* and *P. vivax* DNA from serum samples of microscopically confirmed, malaria-infected patients [17, 18] has demonstrated the feasibility for retrospective diagnosis of malaria infection in specimen banks of cohort studies, such as in determining malaria co-infection in HIV-seropositive populations or arboviruses-infected patients.

In the present study, nested PCR diagnostic assays targeting the 18S rRNA gene of *Plasmodium* species has been used to detect malaria parasite DNA in stored sera. The study revealed the predominance of *P. falciparum* either as single or mixed infections with *P. vivax* or *P. ovale* in Kedougou. The fact that all *P. ovale* infections detected in this study were mixed with *P. falciparum* suggests that *P. ovale* infections might be underestimated by microscopy diagnostic. The high prevalence of *P. falciparum* in Kedougou and other regions in Senegal [16] largely justifies the targeted orientation of malaria control strategies against this species. It was surprising that *P. malariae* failed to be detected since studies in different areas of Senegal [23–25], including Kedougou (Ndiaye et al. unpublished data), have documented the presence of *P. malariae*. The positive amplification obtained with parasite DNA from a microscopy-confirmed *P. malariae*-infected patient in the nested PCR approach rules out a technical issue and undoubtedly confirms the absence of *P. malariae* in the screened samples. A possible explanation of the absence of *P. malariae* in the surveyed samples might be a low parasitaemia below the detection limit of the nested PCR approach. Another explanation might be related to the low prevalence of *P. malariae* in the region. Both *P. ovale* and *P. malariae* have long been reported to be widely distributed in tropical Africa and other major malaria-endemic areas of the world [24, 26–28] and are often associated with *P. falciparum* infections [24, 27] as reported in this study for *P. ovale.*

The present study reports the first substantiated molecular evidence of *P. vivax* infections in patients from Kedougou. There have been other reports of *P. vivax* elsewhere on the African continent where *P. vivax* was initially thought to be absent [6, 7, 11, 13, 14, 29] due to the high prevalence of Duffy-negative individuals considered resistant to *P. vivax* infection [10, 30]. However, reports of documented *P. vivax* presence in West and Central African countries such as Congo [29], Cameroun [14, 15] and Mali [13] highly suggest that this parasite is evolving and adapting, becoming therefore a major public health concern. Infections caused by *P. vivax*, initially thought as ‘benign’ are now gaining higher importance, because of the very wide distribution of *P. vivax* parasite both in tropical and sub-tropical areas [31, 32] and the high number of reported clinical cases [31, 33, 34] along with documented cases of severe *P. vivax* disease and even deaths [35–37]. An important limitation of this study is the inability to link the confirmed *P. vivax* infections with the Duffy phenotypes of the individuals affected, thus the findings reported in the present study deserve to be substantiated by the determination of the Duffy status of the *P. vivax*-infected patients in order to gain insights into the mechanism underlying *P. vivax* infections in Kedougou patients. The inability to link the reported *P. vivax* infections with the disease clinical outcome constitutes an additional limitation of the study. The presence of Duffy-positive ethnic groups that may be present in Senegal, particularly in the Kedougou region, a gold-mining area attracting an important flux of migrants from neighbouring countries, may account for *P. vivax* presence in the area. The recent adaptation of *P. knowlesi*, originally a simian malaria species, to human [2] suggests that a similar adaptation of the *P. vivax* parasite could be occurring, accounting for its increasing prevalence on the African continent.

In recent years, global efforts against malaria have shifted from control to specific strategies aimed at globally eliminating malaria in given areas, implying a need for accurate identification of both *P. falciparum* and non *P. falciparum* spp. that might sustain malaria transmission. In Senegal, the National Malaria Control Programme is already struggling to control malaria due to *P. falciparum*; the additional burden of *P. vivax* infections can therefore be daunting.

## Conclusion

The present study provides the first molecular evidence of *P. vivax* infections in Kedougou (southeastern Senegal). Further investigations with a larger sampling in Kedougou and other Senegalese settings are needed to document the presence and prevalence of *P. vivax* infections in Senegal before orientated strategies can be deployed. The results presented here indicate that there may be a need for the National Malaria Control Programme to anticipate reviewing the management of malaria, including *P. vivax*.
